# Current status of hepatitis C virus infection and countermeasures in South Korea

**DOI:** 10.4178/epih.e2017017

**Published:** 2017-04-13

**Authors:** Sook-Hyang Jeong, Eun Sun Jang, Hwa Young Choi, Kyung-Ah Kim, Wankyo Chung, Moran Ki

**Affiliations:** 1 Department of Internal Medicine, Seoul National University Bundang Hospital, Seoul National University College of Medicine, Seongnam, Korea; 2 Department of Cancer Control and Population Health, Graduate School of Cancer Science and Policy, National Cancer Center, Goyang, Korea; 3 Department of Internal Medicine, Inje University Ilsan Paik Hospital, Goyang, Korea; 4 Graduate School of Public Health, Seoul National University, Seoul, Korea

**Keywords:** Hepatitis C, Epidemiology, Screening, Control, Treatment

## Abstract

Hepatitis C virus (HCV) infection is a major cause of liver cirrhosis, hepatocellular carcinoma, and liver-related mortality. The new antiviral drugs against HCV, direct acting antivirals, result in >90% cure rate. This review aimed to summarize the current prevalence, clinical characteristics, outcomes, and treatment response associated with HCV infection, and countermeasures for optimal HCV control in South Korea. Based on a literature review, the current anti-HCV prevalence in the Korean population is 0.6 to 0.8%, with increasing prevalence according to age. The major HCV genotypes in Korean patients were genotype 1b and genotype 2. Successful antiviral treatment leads to significantly reduced liver related complications and mortality. However, only about one third of the individuals with HCV infection seem to be managed under the current national health insurance system, suggesting a remarkable rate of underdiagnoses and subsequent loss of opportunity to cure. A recent study in South Korea showed that targeted population screening for HCV infection is cost-effective. To prevent recently developed clusters of HCV infection in some clinics, mandatory surveillance rather than sentinel surveillance for HCV infection is required and governmental countermeasures to prevent reuse of syringes or other medical devises, and public education should be maintained. Moreover, one-time screening for a targeted population should be considered and a cost-effectiveness study supporting an optimal screening strategy is warranted.

## INTRODUCTION

**Figure 1.**
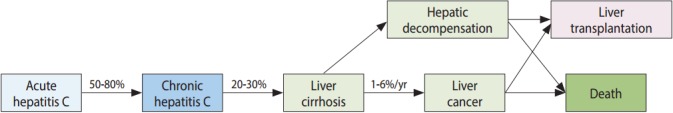
Natural history of hepatitis C virus infection.

Hepatitis C virus (HCV) is a 50 nm-sized enveloped RNA virus that belongs to the family of *Flaviviridae*, and was first discovered in 1989. HCV infection mainly occurs through parenteral routes such as past blood transfusions (before the introduction of a blood screening test for HCV infection in 1991), intravenous drug abuse involving sharing of syringes, and the use of unsterilized medical or non-medical devices. Approximately 170 million persons worldwide have HCV infection. HCV causes acute hepatitis after a 40 to 120-day of incubation period following the initial infection. About 20 to 50% of acute hepatitis C cases spontaneously clear the virus; however, the remaining 50 to 80% will develop chronic HCV infection. If chronic HCV infection is not treated by antiviral therapy, HCV infection is maintained throughout the person’s lifetime, and it may lead to liver cirrhosis, hepatocellular carcinoma, and death from liver disease (Figure 1). Recently, breakthrough antiviral drugs against HCV infection have been developed. Oral antiviral drugs called as direct acting antiviral agents (DAA) with a cure rate of over 90% became available in South Korea (hereafter Korea) since 2015, but the high prices of the drugs is a barrier to treatment of HCV infection.

Recently, clustered outbreaks of HCV infection associated with reuse of disposable syringes were identified in several places. In addition, the diagnosis of HCV infection is not easy because most patients with HCV infection have no or mild symptoms. HCV infection is often diagnosed too late or after its complications such as liver cirrhosis or hepatocellular carcinoma have manifested because there is a low awareness of HCV infection among healthcare professionals and the general public. An effective vaccine against HCV infection has not been developed thus far. In this article, the current status of HCV infection and its countermeasures in Korea are reviewed.

## MAIN BODY

### Prevalence of hepatitis C virus infection in Korea

The age, sex, and region-adjusted prevalence of anti-HCV was 0.78% in 291,314 adults aged 20 years or older who underwent health examinations in 2009. The male to female ratio in those with anti-HCV antibody positivity was 1:1. However, the prevalence of anti-HCV increased with age from 0.6% in health examinees aged 40-49 years to 0.8% in those aged 50-59, 1.5% in those aged 60-69, and 2.3% in those aged 70 years or older. The prevalence of anti-HCV was significantly higher in Busan, Gyeongnam, and Jeonnam, and was significantly lower in Jeju compared to other regions, showing a regional difference. Of 1,718 anti-HCV-positive individuals, 478 underwent HCV RNA tests, and rate of HCV RNA positivity was 56.1% [1].

Of 17,764 subjects of the Korea National Health and Nutrition Examination Survey 2012–2014, 140 subjects were found to be anti-HCV-positive, and the prevalence of anti-HCV was 0.62% (95% confidence interval [CI], 0.49 to 0.78%) in those aged 10 years or older and 0.68% (95% CI, 0.54 to 0.86%) in those aged 20 years or older. The prevalence of anti-HCV was higher in females (male to female ratio 1.38) and increased with age in both males and females. The prevalence of anti-HCV was significantly high in Busan, Gyeongnam, Ulsan, and Chungbuk, and was significantly low in Jeju compared to other regions. The positive rate of HCV RNA in those who were positive for anti-HCV was 32.5%.

According to a study that analyzed the number of adult patients aged 20 years or older who received treatment for HCV infection as a diagnostic disease (acute HCV infection [B17.1] or chronic HCV infection [B18.2] as a primary disease and a secondary disease) using the 2005-2012 data from the National Health Insurance Corporation, the number of patients with HCV infection was 52,515 in 2005, 68,543 in 2009, and 73,502 in 2012. The prevalence of managed patients with HCV infection under National Insurance, which was calculated based on the registered standard population in 2010, was 0.14% in 2005, 0.18% in 2009, and 0.18% in 2012. The prevalence of HCV infection increased with age and was slightly higher in males (male to female ratio 0.95). These data also revealed that the prevalence rates of HCV infection were significantly higher at 0.35% in Busan, 0.32% in Jeonnam, and 0.24% in Gyeongnam compared to other regions. Interestingly, the increase in the prevalence of HCV infection was fastest in Jeju from across the country (0.15% in 2005 to 0.23% in 2012) [2].

In summary, the prevalence of anti-HCV-positive patients among Korean adults aged 20 years or older is 0.6 to 0.8%, and the prevalence rates in Busan, Gyeongnam, and Jeonnam are high. About 1/2 to 1/3 of anti-HCV positive patients are viremic and thus are candidates for antiviral treatment. Those without viremia are assumed to have recovered spontaneously from HCV infection in the past or were cured by treatment with interferon-based antiviral therapy or a small number may be a false-positive result for anti-HCV. Meanwhile, in view of the fact that the prevalence of patients who were treated for a diagnostic code such as HCV infection in medical facilities was reported to be less than 0.2%, it is postulated that only approximately 25 to 35% of all patients with HCV infected patients visited healthcare systems, whereas the remaining 65 to 75% are undiagnosed and not aware of their HCV infection (Table 1). Therefore, it is necessary to consider the usefulness of screening for HCV infection for a targeted population based on age because of the increased prevalence of HCV infection.

### Risk factors and clinical characteristics of patients with hepatitis C virus infection in Korea

The results of a HCV cohort study of 1,173 patients with HCV infection who were diagnosed at five university hospitals from 2007 to 2011 in Korea found that the risk factors for HCV infection (independent factors showing significant differences in the group of patients with HCV infection compared to the control group of 531 liver disease patients without hepatitis B virus (HBV) and HCV infection) were age, past blood transfusion, intravenous drug abuse, needle stick injury, sexual relationships with more than three partners, tattoos, and experience of diagnostic endoscopy. In that study, the proportion of those with experience of intravenous drug abuse among all patients with HCV infection was found to be 5%, which was much lower than that in western patients, and in most cases, past invasive procedures were found to be a major risk factor for HCV infection [3].

In a study involving 234 age-matched and sex-matched HCV infection case and the HBV infection control and healthy control pairs who visited university hospitals in Gyeongnam and Jeonnam areas with a high prevalence of HCV in 2013, risk factors for HCV infection were compared between the case group and the control groups. The results showed that the rate of sharing razors was significantly higher in HCV infection case at 2.39-fold and 3.29-fold higher compared with HBV infection controls and healthy controls, respectively, and that the rate of having more than four sexual partners was significantly higher in HCV infection cases, 2.15-fold and 6.89-fold higher compared with HBV infection controls and healthy control persons, respectively. The rates of contact with dockworkers and tattoos in HCV infection cases were 1.91-fold and 2.20-fold higher, respectively, compared to controls of HBV infection and healthy controls. The rates of blood transfusion, history of invasive operations, piercings, and a history of acupuncture in HCV infection cases were significantly higher at 5.38-fold, 5.02-fold, 5.95-fold, and 2.08-fold, respectively, when compared with healthy controls [4].

The mean age of patients with HCV infection was 55 years, and the ratio of male to female was about 1. The proportion of past and current drinkers accounted for 53.4% of the study population, of whom 3.0% were coinfected with HBV. The common comorbidities included cardiovascular (24.8%), diabetes (14.7%), thyroid disease (6.0%), psychiatric illness (5.8%), and renal disease (3.6%). Liver biopsy was performed in 301 patients (25.7%). The liver biopsy results showed that the proportions of those with a Metavir grade of F0, F1, F2, F3, and F4 in terms of degree of liver fibrosis were 7.5, 28.6, 32.7, 18.8, and 12.4%, respectively. The proportion of patients with Metavir grade 3 and 4 who were considered to have advanced liver fibrosis requiring urgent treatment accounted for about 31.0%. In terms of diagnostic categories, the proportions of acute hepatitis, past infection, chronic hepatitis, liver cirrhosis, and liver cancer were 5.3, 3.2, 66.2, 15.3, and 10.0%, respectively. The major HCV genotypes were genotype 1b (45%) and genotype 2 (45%) [3].

### Natural history and interferon-based treatment response in Korean patients with hepatitis C virus infection

**Figure 2.**
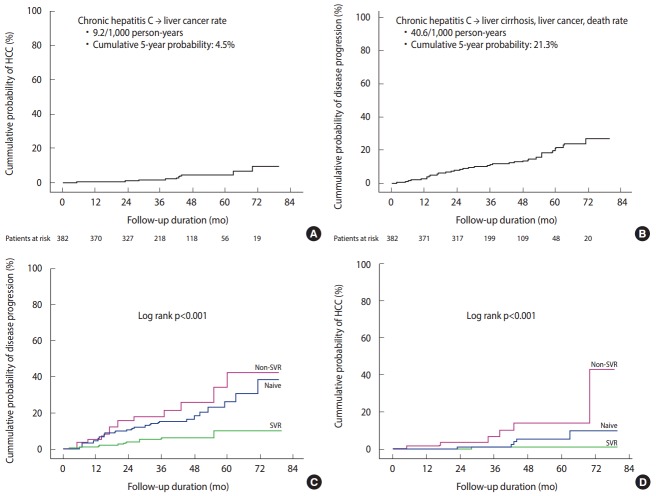
The prospective clinical outcomes of chronic hepatitis C patients in Korea (A and B) [5]. The difference of clinical outcomes of chronic hepatitis C patients among treatment-naive patients (Naive), those who did not achieve a sustained virologic response (Non-SVR), and those with a sustained virologic response (SVR). (C) The composite disease progression rate including development of liver cirrhosis, decompensation, liver cancer, and mortality was lowest in the “SVR” group compared to the “Non-SVR” or “Naive” group. (D) The cumulative probability of liver cancer development was lowest in the “SVR” group compared to the “Non-SVR” or the “Naive” group [5]. HCC, hepatocellular carcinoma.

In a Korean HCV cohort study of 382 HCV RNA-positive patients with chronic HCV infection (mean age, 55 years) who were newly diagnosed with HCV infection at six university hospitals in 2007-2012 and were not previously treated, the subjects were prospectively followed-up for 39 months. The results showed that the rate of patients with chronic hepatitis who developed liver cirrhosis, decompensated cirrhosis, and hepatocellular carcinoma were 11.0, 1.0, and 3.1%, respectively, and the cumulative probability of developing cirrhosis at five years was 16.7% and that of developing hepatocellular carcinoma at five years was 4.5%. The incidence of liver cirrhosis in patients with chronic HCV infection was 33.0/1,000 person-years, while the incidence of hepatocellular carcinoma in patients with chronic HCV infection was 9.2/1,000 personyears (Figure 2). The overall 3-year and 5-year survival rates in these patients was good at 99.7 and 96.0%, respectively. The interferon-based antiviral treatment was administered to 62.0% of the patients, and the rate of sustained virologic response (SVR) indicative of a cure was 74.3% in the treated patients, showing good outcomes. The independent factors for poor clinical outcome were age > 55 years, low platelet count, and failure to achieve a SVR. Overall, the prognosis of chronic HCV infection was good, but if liver disease progressed and antiviral therapy failed, the prognosis was poor (Figure 2) [5].

According to the results of a Korean HCV cohort study in which 196 patients with HCV RNA-positive liver cirrhosis (mean age, 61 years) were prospectively followed-up for 39 months at six university hospitals from 2007 to 2012, 15.8% of the subjects developed hepatocellular carcinoma and 16.8% died or underwent liver transplantation. The estimated incidence of hepatocellular carcinoma in patients with liver cirrhosis associated with HCV infection in Korea was 5.8/100 person-years and the overall mortality rate was 5.1/100 person-years. The 3-year cumulative incidence of hepatocellular carcinoma was 19.1% and the 3-year cumulative mortality rate was 14.5%. The results of a multivariable analysis showed that the independent factors for the development of hepatocellular carcinoma were the absence of anti-HBV surface antibody and reduced serum albumin levels, while the independent factors for the related mortality rate were the presence of ascites, reduced albumin levels, and non-achievement of SVR. The incidence of hepatocellular carcinoma related to liver cirrhosis is considered high around the world, and it could be that as liver disease progresses and antiviral therapy fails, it is associated with a poor prognosis [6].

The cure rate or SVR rate in patients with chronic HCV infection who were treated with antiviral therapy between 2000 and 2008 at 14 university hospitals in Gyeonggi and Incheon area was 53.6% for HCV genotype 1 and 71.4% for genotype 2 or 3, and the overall cure rate was approximately 60.0% regardless of genotype. According to the results of a Korean HCV cohort study of 759 patients without a history of past treatment conducted in 2007-2013, it was found that interferon-based antiviral treatment (a combination of peginterferon alpha plus ribavirin given for 48 weeks for genotype 1 or for 24 weeks for genotype 2) was undertaken in 37.3% of all patients and the 5-year cumulative treatment rate was 39.4%, indicating that about 2/3 patients were not treated with the combination therapy due to associated adverse events or contraindications [7]. This treatment rate was based on the results found in university hospitals where patients and doctors were actively involved in antiviral therapy. Meanwhile, according to the results of an analysis of data from the National Health Insurance Review Agency in 2009-2013, only 35,006 (10.3%) out of a total of 340,756 patients with HCV infection were treated with interferon-based antiviral therapy. Therefore, it was estimated that the actual antiviral treatment rate across the country is less than 1/3 of that in university hospitals [8].

### Introduction and effects of oral antiviral therapies for hepatitis C virus

The combination therapy with subcutaneous peginterferon and oral ribavirin was a non-specific antiviral treatment for HCV, whereas antivirals introduced since 2014 are HCV-specific DAA drugs that directly interfere with the major steps of HCV replication based on the life cycle of HCV. Table 2 summarizes DAA drugs that have been approved since 2015 in Korea and became reimbursable under the health insurance system after 2016.

When DAAs are orally administered for 12-24 weeks, the SVR is over 90% and they have an excellent safety profile. DAA drugs can be used in almost all patients with HCV infection who are not eligible for conventional interferon-based treatment [9]. Although the idea of eradicating HCV by DAA treatment has become a reality, the very high price of the drugs, which puts financial burdens on the health insurance budgets and patients, and cautions regarding drug drug interactions, are potential impediments. As mentioned in the results of prevalence studies related to HCV infection in Korea, the fact that there may be many undiagnosed patients becomes an important topic about the current management of HCV in Korea.

### Status of national control system for hepatitis C virus infection

According to the Infectious Disease Control and Prevention Act prior to its revision at the end of 2016, HCV infection was classified as a designated infectious disease that is required to be monitored and investigated for its outbreaks. The national sentinel surveillance system was in operation, under which hospitals nationwide in 2001-2010 and a hospital per 200,000 population from 2011 (186 hospitals, 2016) were designated as sentinel surveillance institutions to notify and report HCV infection [10]. However, in view of the fact that the recent outbreaks of HCV infection occurred mainly in clinics and were not monitored via the sentinel surveillance system, in September 2016 the Ministry of Health and Welfare announced a strategy for the prevention and countermeasures of HCV infection in that the sentinel surveillance system will be switched to an exhaustive surveillance system of infectious disease. Therefore, the status of HCV infection was changed from designated infectious disease to Group 3 nationally notifiable infectious disease. The revised Infectious Disease Control and Prevention Act will be effective from June 3, 2017, and active control of infectious disease will thus be needed through exhaustive surveillance.

### Necessity and cost-effectiveness of screening programs for hepatitis C virus

According to the results of a study estimating the direct medical costs of managing chronic HCV in 445 patients with HCV infection who visited eight university hospitals between 2011 and 2012, it was found that as the liver disease state progressed from chronic HCV infection to liver cirrhosis, uncompensated liver cirrhosis, and liver cancer, the mean direct medical costs per month for each state increased from USD 183 to 252, 1,020, and 1,375, respectively. Approximately 72% of the direct medical costs were reimbursed under the National Health Insurance and the remaining 28% were paid by patients themselves. The mean number of outpatient visits was 7.01, 7.36, 8.30, and 11.56 days for each state, while the number of hospitalization days was 0.19, 0.77, 8.75, and 12.86 days for each state [11]. When comparing mean medical costs per month between chronic HCV patients who were cured with antiviral treatment (n= 69) and those who were not cured with antiviral treatment (n= 215), the results showed that the mean monthly medical costs for those who were cured was USD 76, whereas the costs for those who were not cured was USD 490, a large difference [11]. Therefore, treating chronic HCV infection before its progression to liver cirrhosis or liver cancer is the most cost-effective in a long-term perspective.

**Figure 3.**
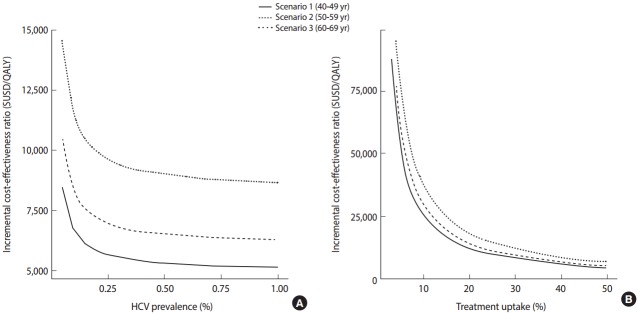
The cost-effectiveness analysis of anti-HCV screening for Korean population in their 5th, 6th, and 7th decade, respectively, with various prevalence (A) and treatment rates (B) [10]. QALY, quality adjusted life years.

Meanwhile, in view of the fact that antiviral treatment is only administered after patients are diagnosed with chronic HCV infection and there is evidence that there exist many undiagnosed patients, the need for screening for HCV infection among the Korean population has emerged. In the US, a screening program for HCV is being conducted according to the year of birth based on the findings of studies indicating that a screening strategy for a targeted population with a high prevalence of anti-HCV is cost-effective. In Korea, the prevalence of HCV infection increases with age. The results of a study investigating the cost-effectiveness of a one-time HCV screening of a population in their 40s, 50s, or 60s and subsequent treatment with DAA consistent with the current reimbursement guideline in comparison with no screening are summarized as follows. Using this screening and treatment strategy, a total of 43,635 undiagnosed patients were estimated to be identified and 17,193 patients were estimated to receive treatment. When populations aged 40s, 50s, or 60s were screened, the estimated incremental cost-effectiveness ratios (ICERs) for each age group were USD 5,714 (Korean won [KRW] 8.4 million), USD 6,843 (KRW 10.76 million), and USD 8,889 (KRW 15.89 million) per quality-adjusted life year gained, respectively (Figure 3). Assuming that the willingness-to-pay threshold (gross domestic product per capita 2015) is USD 27,512, which is generally applied in Korea, a one-time anti-HCV screening for those in their 40s to 60s was cost-effective and, in particular, was the most cost-effective for those in their 40s [12].

In Korea, there is a health screening system that encompasses the entire population, and the number of screening institutions is 8,576 as of the end of 2012. In 2012, the general health examination rate was 72.9% and the cancer screening rate was 39.4%. The screening costs paid by the National Health Insurance were about KRW 967.2 billion. Although the current incidence of HCV infection in Korea is not known, HCV RNA testing is performed in blood transfusion screening, and the incidence of HCV infection due to intravenous drug abuse is still low. The incidence of HCV infection is expected to be very low in the future if the recently established national control system works well, in particular with regard to the outbreaks of HCV infection in some medical institutions. Therefore, if evidence supporting economic evaluation of HCV screening strategy become available in the future, it is worth considering that a one-time anti-HCV test should be temporarily added to general health examination items for the targeted population aged 40–65 years, and afterwards anti-HCV test is to be included in the national health screening for transitional ages (40, and 66 years). It was reported in the media that the Ministry of Health and Welfare announced a strategy for the prevention and countermeasure of HCV infection on September 6, 2016 and that HCV screening tests will be conducted as a pilot program for the examinees of the national health screening for transitional ages in the areas with a high prevalence of HCV infection.

## CONCLUSION

Currently, the prevalence of HCV infection in Korea is less than 1% (0.6-0.8%). Because HCV RNA testing is performed in transfusion screening in Korea, HCV infection due to blood transfusion is not expected to occur in the future. However, recent outbreaks of HCV infection in health care facilities have become a public health problem that requires a national response. It is necessary to implement strict quality control of invasive procedures in medical institutions and non-medical institutions at the national level and to increase public awareness of the use of disposable syringes through media publicities. Although the incidence of HCV infection due to intravenous drug abuse is still low, it is necessary to continue government-level countermeasures to prevent this. If integrated preventive measures for the aforementioned items are implemented, the incidence of HCV infection in Korea can be lowered.

Since 2016, safe and effective antiviral drugs that can cure HCV infection have been covered by the national health insurance system, and effective treatment with such antiviral drugs has already begun in diagnosed patients. However, HCV infection is asymptomatic. Therefore, if HCV screening tests in conjunction with the national health examination is performed for undiagnosed patients with progressing liver disease, the treatment and diagnosis costs will increase in the short term, but it will be a cost-effective HCV eradication strategy to increase the quality of life and reduce mortality in the long term. We hope that Korea will be achieving complete eradication of HCV infection the earliest in the world through timely actions and management strategies.

## Figures and Tables

**Table 1.** The prevalence of hepatitis C virus (HCV) infection in Korea

Year	Subjects	No. of subjects	Anti-HCV positivity (%)	HCV RNA positivity (%)
2009	Nationwide 29 health check centers	291,314	0.78 (aged ≥ 20 yr)	56.1
2012-2014	National Health and Nutrition Examination Survey	17,764	0.62 (aged ≥ 10 yr)	32.5
			0.68 (aged ≥ 20 yr)	
2005-2012	National Health Insurance Service data, subjects with diagnostic code of acute or chronic HCV infection	52,512 (2005)	0.14 (2005)	
		68,543 (2009)	0.18 (2009)	
		73,502 (2012)	0.18 (2012)	
			(aged ≥ 20 yr)	

**Table 2.** Direct acting antiviral drugs against hepatitis C virus infection currently approved in Korea

Drug class	Drug	Manufacturer	Approved in Korea	
NS3/4A protease inhibitor	Asunaprevir	BMS	2015	Combination with daclatasvir
NS5A inhibitor	Daclatasvir	BMS	2015	Combination with asunaprevir or sofosbuvir
	Ledipasvir	Gilead	2015	Combination with sofosbuvir
NS5B inhibitor	Sofosbuvir	Gilead	2015	
NS5A/5B inhibitor	Ledipasvir/sofosbuvir	Gilead	2015	Single tablet complex
