# Supplementary material for: Current status of hepatitis C virus infection and countermeasures in South Korea
Source: Epidemiol Health. 2017 Apr 13;39:e2017017. doi: 10.4178/epih.e2017017 (PMC5543292; doi:10.4178/epih.e2017017)
Supplement: Supplementary file 1 [file epih-39-e2017017-supplementary.pdf]

Open Access

Volume: 39, Article ID: e2017017, 7 pages
https://doi.org/10.4178/epih.e2017017

epiH Epidemiology and Health

EDITORIAL

한국의 최근 C형간염 현황과 대책

정숙향[1], 장은선[1], 최화영[2], 김경아[3], 정완교[4], 기모란[2]

[1]서울대학교 의과대학 분당서울대학교병원 내과; [2]국립암센터 국제암대학원대학교 암관리학과; [3]인제대학교 일산 백병원 내과; [4]서울대학교 보건대학원

Current Status of Hepatitis C Virus Infection and Countermeasures in South Korea

Sook-Hyang Jeong[1], Eun Sun Jang[1], Hwa Young Choi[2], Kyung-Ah Kim[3], Wankyo Chung[4], Moran Ki[2]

[1]Department of Internal Medicine, Seoul National University Bundang Hospital, College of Medicine, Seoul National University, Seongnam, Korea; [2]Department of Cancer Control and Population Health, Graduate School of Cancer Science and Policy, National Cancer Center, Goyang, Korea; [3]Departments of Internal Medicine, Inje University Ilsan Paik Hospital, Goyang, Korea; [4]Graduate School of Public Health, Seoul National University, Seoul, Korea

KOREAN SUMMARY

우리나라 C형간염 유병률은 1% 미만(0.6–0.8%)이며, 수혈에 의한 C형간염은 더 이상 발생하지 않을 것으로 보인다. 그러나 최근 의료기관에서 C형간염 집단감염이 발생하여 국가적 대응이 필요한 공중보건의 문제로 떠올랐다. 2016년 부터는 C형간염을 완치할 수 있는 효과적이고 안전한 약제가 건강보험급여로 인정되어 이미 진단된 환자들에서는 효과적인 치료가 시작되었다. 그러나 진단되지 않은 상태에서 간질환이 진행되고 있는 환자들을 발굴하기 위해 국가 검진체계와 연계하여 선별검사를 시행한다면, 단기적으로는 진단과 치료비용 부담이 커지겠지만 장기적으로 사망률을 감소시키고, 삶의 질을 높이는 비용효과적인 C형간염 퇴치 전략이 될 것이다.

Hepatitis C virus (HCV) infection is a major cause of liver cirrhosis, hepatocellular carcinoma (HCC), and liver-related mortality. The new antiviral drugs, direct acting antivirals (DAA) against HCV, leads to more than 90% of cure rate. This review aimed to overview the current prevalence, clinical characteristics and outcomes with treatment response, and countermeasures for optimal HCV control in South Korea. 1. By way of literature review, current anti-HCV prevalence in Korean population is 0.6-0.8%, with increasing prevalence according to the age. The major HCV genotypes in Korean patients were genotype 1b and genotype 2. Successful antiviral treatment leads to significantly reduced liver related complications and mortality. However, only about one third of the infected people seemed to be managed under current national health insurance system, suggesting a remarkable underdiagnoses and subsequent loss of opportunity to cure. A recent study showed that targeted population screening of HCV infection is cost-effective in South Korea. 2. To prevent recently developed several cluster HCV infection in some clinics, mandatory surveillance rather than sentinel surveillance for HCV infection is required, and governmental countermeasures to prevent reuse of syringes or other medical devises and public education should be maintained. Moreover, one-time screening for targeted population should be considered and cost-effectiveness study supporting optimal screening strategy is warranted.

KEY WORDS: Hepatitis C, Epidemiology, Screening, Control, Treatment

Correspondence: Moran Ki
Department of Cancer Control and Population Health, Graduate School of Cancer Science and Policy, National Cancer Center, 323 Ilsan-ro, Ilsandong-gu, Goyang 10408, Korea
E-mail: moranki@ncc.re.kr

이 원고는 2017년 3월 2일 주간건강과 질병 제10권 제9호에 '국내 C형간염의 현황과 대책'(Current Status of Hepatitis C Virus Infection and Countermeasures' 제목으로 수록된 것을 수정보완한 것입니다.

Received: Mar 25, 2017 / Accepted: Apr 13, 2017 / Published: Apr 13, 2017

This article is available from: http://e-epih.org/

This is an open-access article distributed under the terms of the Creative Commons Attribution License (http://creativecommons.org/licenses/by/4.0/), which permits unrestricted use, distribution, and reproduction in any medium, provided the original work is properly cited.

© 2017, Korean Society of Epidemiology

서 론

C형간염바이러스(hepatitis C virus, HCV)는 50 nm 크기의 외피를 가진 RNA 바이러스로 *Flaviviridae* family에 속하며 1989년에 그 존재가 처음 밝혀졌다. 주로 C형간염 선별검사 이전(1991년 이전) 혈액제제로 수혈 받거나, 주사기를 공유하는 정맥주사 약물 남용자, 멸균되지 않은 의료기기 사용 등의 혈액 전파 경로로 감염이 일어나는데 전 세계적으로 약 1억 7천만 명이 감염되어 있다. HCV 감염 후 약 40-120일의 잠복기를 거쳐 급성 C형간염을 일으키는데 감염자 중 약 20-50%는 저절로 회복되지만, 50-80%는 만성간염으로 이행한다. 만성간염이 되면 항바이러스 치료로 완치시키지 못할 경우 평생 HCV 감염상태가 유지되며, 간경변증 및 간세포암이 발생할 수 있고 이로 인하여 간질환 사망이 초래된다 (Figure 1). C형간염의 치료제는 최근 획기적으로 발전되어, 완치율 90% 이상의 경구 항바이러스 치료제들(direct antiviral agents, DAA)이 2015년부터 국내에서 사용되고 있으나, 고가의 약제비가 치료 장벽이 되고 있다.

최근 우리나라에서는 1회용 주사기 재사용 등에 의한 C형간염의 집단 발생이 여러 곳에서 확인되었다. 또한 대부분 C형간염 환자들은 증상이 없거나 미미하여 진단이 쉽게 되지 않고, 아직 의료진이나 일반인에서 C형간염에 대한 인지도가 낮아서 간경변증이나 간암의 합병증이 생긴 후에 즉, 너무 늦게 진단되는 경우가 많다. C형간염을 사전에 예방할 수 있는 효과적인 백신은 아직 개발이 되지 않았고, 그 전망도 밝지 않은 실정이다. 이 글에서는 현재 국내 C형간염의 현황과 관리대책에 대해 고찰해 보고자 한다.

몸 말

국내 C형간염바이러스 유병률

2009년 국내 20세 이상 성인 건강검진자 291,314명에서 나이, 성별, 지역보정 anti-HCV 유병률은 0.78%였다. 남녀비는 1.1로 비슷하지만 나이가 들수록 anti-HCV 유병률도 증가하여 40-49세 검진자에서는 0.6%, 50-59세에서는 0.8%, 60-69세에서는 1.5%, 70세 이상에서는 2.3%로 높아졌다. 다른 지역에 비해 부산, 경남, 전남이 유의하게 높고 제주는 유의하게 낮은 유병률을 보여 지역적인 차이가 관찰되었다. Anti-HCV 양성자 1,718명 중에서 HCV RNA 검사가 시행된 경우는 478명이었고, 이들에서 HCV RNA 양성률은 56.1%였다[1].

2012-2014년 국민건강영양조사 17,764명 대상자 중에서는 140명에서 anti-HCV 양성률을 보였는데, 10세 이상 인구에서 anti-HCV 유병률은 0.62% (95% 신뢰구간, 0.49-0.78%), 20세 이상 인구에서는 0.68% (95% 신뢰구간, 0.54-0.86%)였다. 남녀비는 1.38로 여성에서 유병률이 높았고 나이가 증가함에 따라 유병률이 증가하는 추세는 동일하였다. 또 부산, 경남, 울산, 충북이 다른 지역에 비해 유병률이 유의하게 높았고 제주지역은 유의하게 낮았다. anti-HCV 양성자에서 HCV RNA 양성률은 32.5%였다.

2005-2012년 건강보험공단 자료를 이용하여 C형간염 상병명으로[주상병 및 부상병에 급성 C형간염(B17.1) 및 만성 C형간염(B18.2)] 진료받은 20세 이상 성인 환자수를 분석한 연구 결과에 의하면 환자수는 2005년 52,515명, 2009년 68,543명 및 2012년 73,502명이었고, 2010년 국내표준인구를 사용하여 계산한 진료받은 환자 유병률은 각각 0.14%, 0.18% 및 0.18%였다. 인구의 나이 증가에 따라 유병률이 증가하고 남녀비는 0.95로 남자환자가 약간 많았다. 이 자료에서도 부산, 전남, 경남의 환자 유병률이 각각 0.35%, 0.32%, 0.24%로 다른 지역에 비해 유의하게 높았다. 한편 제주의 C형간염 유병률은 전국에서 가장 빠르게 증가하고 있음이 특이하였다(2005년 0.15% → 2012년 0.23%) [2].

요약하면 국내 20세 이상 성인에서 C형간염 항체 유병률은 0.6-0.8% 정도이고, 부산, 경남, 전남의 유병률이 높은 특성을 보인다. 항체양성자 중 1/2-1/3 에서 바이러스 혈증(viremia)을 동반하여 항바이러스 치료대상자가 되고, 바이러스 혈증을 보이지 않는 사람들은 과거 감염에서 저절로 회복되었거나 과거 인터페론 기반의 항바이러스 치료로 완치된 환자들 또는 소수의 항체 위양성 반응자들로 짐작된다. 한편 실제로 의료기관에서 C형간염 상병명으로 진료를 받은 환자 유병률은 0.2% 미만이므로, 전체 C형간염 감염자의 약 1/3-1/4만이 진료를 받았으며, 2/3-3/4의 환자들은 진단되지 않은 채 질병을 인식하지 못하고 있을 것으로 추정된다(Table 1). 따라서 C형간염 유병률이 증가하는 나이의(40대 또는 50대 이상) 인구집단을 목표로 한 선별검사의 유용성을 고려해야 한다.

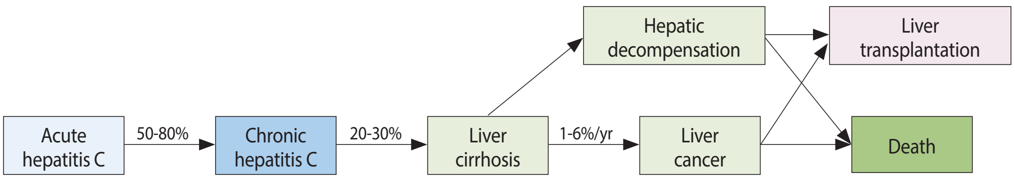

Figure 1. Natural history of hepatitis C virus infection.

Table 1. The prevalence of hepatitis C virus (HCV) infection in Korea

Year	Subjects	No. of subjects	Anti-HCV positivity (%)	HCV RNA positivity (%)
2009	Nationwide 29 health check centers	291,314	0.78 (aged ≥20 yr)	56.1
2012-2014	National Health and Nutrition Examination Survey	17,764	0.62 (aged ≥10 yr)<br>0.68 (aged ≥20 yr)	32.5
2005-2012	National Health Insurance Service data, subjects with diagnostic code of acute or chronic HCV infection	52,512 (2005)<br>68,543 (2009)<br>73,502 (2012)	0.14 (2005)<br>0.18 (2009)<br>0.18 (2012)<br>(aged ≥20 yr)	

국내환자군의 감염 위험인자 및 임상적 특징

2007-2011년 국내 5개 대학병원에서 진단된 C형간염 환자 1,173명을 대상으로 한 HCV 코호트 연구 결과 밝혀진 C형 간염의 감염위험요인은 나이, 과거 수혈, 정맥주사 약제남용, 주사바늘에 찔린 경험, 3명 이상의 성상대자를 가진 경우, 문신 및 내시경 경험이었다(대조군은 B형간염과 C형간염이 모두 없는 간질환 환자군 531명이었음). 전체 C형간염 환자군에서 정맥주사 약제남용의 경험자는 5%로 서구 환자군에 비해 매우 낮았으며, 대부분 환자들에서 과거 침습적 시술이 C형간염의 주요 위험인자였음을 알 수 있었다[3].

2013년 국내 HCV 고유병 지역인 경남과 전남의 대학병원에 방문한 HCV환자 234명을 대상으로 이 지역에서 HCV 감염 요인을 분석한 다른 연구에 의하면(환자군과 연령 및 성을 짝짓기 하여 HBV환자 1명과 HCV와 HBV가 모두 없는 건강한 사람 1명을 각각 대조군으로 선정하여 HCV위험요인을 비교 분석함) HCV환자는 B형 간염환자와 건강한 사람 대비 면도기 공동사용이 각각 2.39배, 3.29배 유의하게 높았고, 4명 이상의 성 상대자는 각각 2.15배, 6.89배 유의하게 높았다. 또한 B형 간염 환자에 비하여 항만근로자 접촉은 1.91배 높았고, 문신은 2.20배 높았다. 건강한 사람에 비하여 수혈은 5.38배 높았고, 출혈이 있는 수술 과거력도 5.02배 높았으며, 피어싱은 5.95배, 침을 맞은 과거력은 2.08배 유의하게 높게 나타났다[4].

국내 C형간염 환자들의 나이는 평균 55세, 남녀비는 비슷하였고, 과거 및 현재 음주자가 53.4%를 차지하였다. 이들 중 약 3.0%에서 B형간염과 중복감염이 있었으며, 흔한 동반 상병으로는 심혈관질환(24.8%), 당뇨병(14.7%), 갑상선질환(6.0%), 정신과질환(5.8%), 신장질환(3.6%)였다. 간생검은 301명(25.7%)에서 시행되었는데 그 결과 간 섬유화(liver fibrosis) 정도는 Metavir 등급 0, 1, 2, 3, 4단계가 각각 7.5%, 28.6%, 32.7%, 18.8%, 12.4%였다. 즉, 치료의 시급성을 요하는 진행된 간섬유화로 간주되는 3, 4단계의 환자가 약 31.0%를 차지하였다. 진단 구분으로는 급성간염, 과거 감염, 만성간염, 간경변증, 간암이 각각 5.3%, 3.2%, 66.2%, 15.3%, 10.0%를 차지하였다. 주요 HCV 유전자형은 genotype 1b (45%)와 genotype 2 (45%)이었다[3].

국내 C형간염 환자들의 자연경과와 인터페론 기반 치료 반응

2007-2012년 6개 대학병원에서 처음으로 진단되고 과거 치료받은 적이 없으며 HCV RNA가 양성인 만성 C형간염환자 382명을 대상으로(평균 나이 55세), 전향적으로 39개월을 관찰한 한국 HCV 코호트 연구결과에 의하면 만성간염에서 간경변증 및 비대상성 간경변증, 간세포암으로 진행한 환자들은 각각 11.0%, 1.0%, 3.1%였으며, 5년 누적 간경변증 발생률은 16.7%, 5년 누적 간세포암 발생률은 4.5%였다. 만성C형간염에서 간경변증 발생률은 33.0/1,000 person-years, 간세포암 발생률은 9.2/1,000 person-years였다(Figure 2). 이들 환자군의 3년, 5년 생존율은 각각 99.7%, 96.0%로 좋았으며 인터페론 기반의 항바이러스 치료는 62.0%에서 시행되었고 완치를 나타내는 지속바이러스반응(sustained virologic response, SVR)은 74.3%에 도달하여 좋은 치료성적을 보여주었다. 나쁜 임상경과를 예측할 수 있는 독립인자로는 나이 > 55세, 혈소판수치 감소, SVR에 도달하지 못함으로 나타났다. 전반적으로 만성 C형간염의 예후는 좋지만 간질환이 진행할수록 또 항바이러스 치료에 실패할수록 나쁜 예후와 관련됨을 알 수 있었다(Figure 2) [5].

2007-2012년 6개 대학병원에서 HCV RNA가 양성인 간경변증 환자 196명을 대상으로(평균나이 61세) 전향적으로 39개월을 관찰한 한국 HCV 코호트 연구결과에 의하면 15.8%에서 간세포암이 발생하고 16.8%에서 사망하거나 간이식을 받았다. 국내 C형간염에 의한 간경변증 환자에서 간세포암 발생률은 5.8/100 person-years, 사망률은 5.1/100 person-years 였고, 3년 누적 간세포암 발생률은 19.1%, 3년 누적 사망률은 14.5%였다. 다변량 분석 결과 간세포암 발생과 관련된 독립인자로는 B형간염 항체가 없는 경우와 혈청 알부민치 저하였으며, 사망률과 관련된 독립인자로는 복수, 혈청 알부민치 저하, SVR에 도달하지 못함이었다. 이러한 간경변증에서 간세포암 발생률은 전세계적으로 높은 것으로 여겨지며, 진행된 간질환 또는 항바이러스 치료실패가 나쁜 예후와 관련있음을 확인할 수 있었다[6].

2000-2008년동안 경인지역 14개 대학병원에서 항바이러스 치료를 받은 만성 C형간염 환자들의 완치율은 HCV genotype 1에서는 53.6%, genotype 2 또는 3에서는 71.4%로, 전체적으로 약 60.0%의 완치율을 보여주었다. 2007-2013년 과거 치료력이 없는 759명의 환자들을 대상으로 한 한국 HCV 코호트 연구결과에 의하면 인

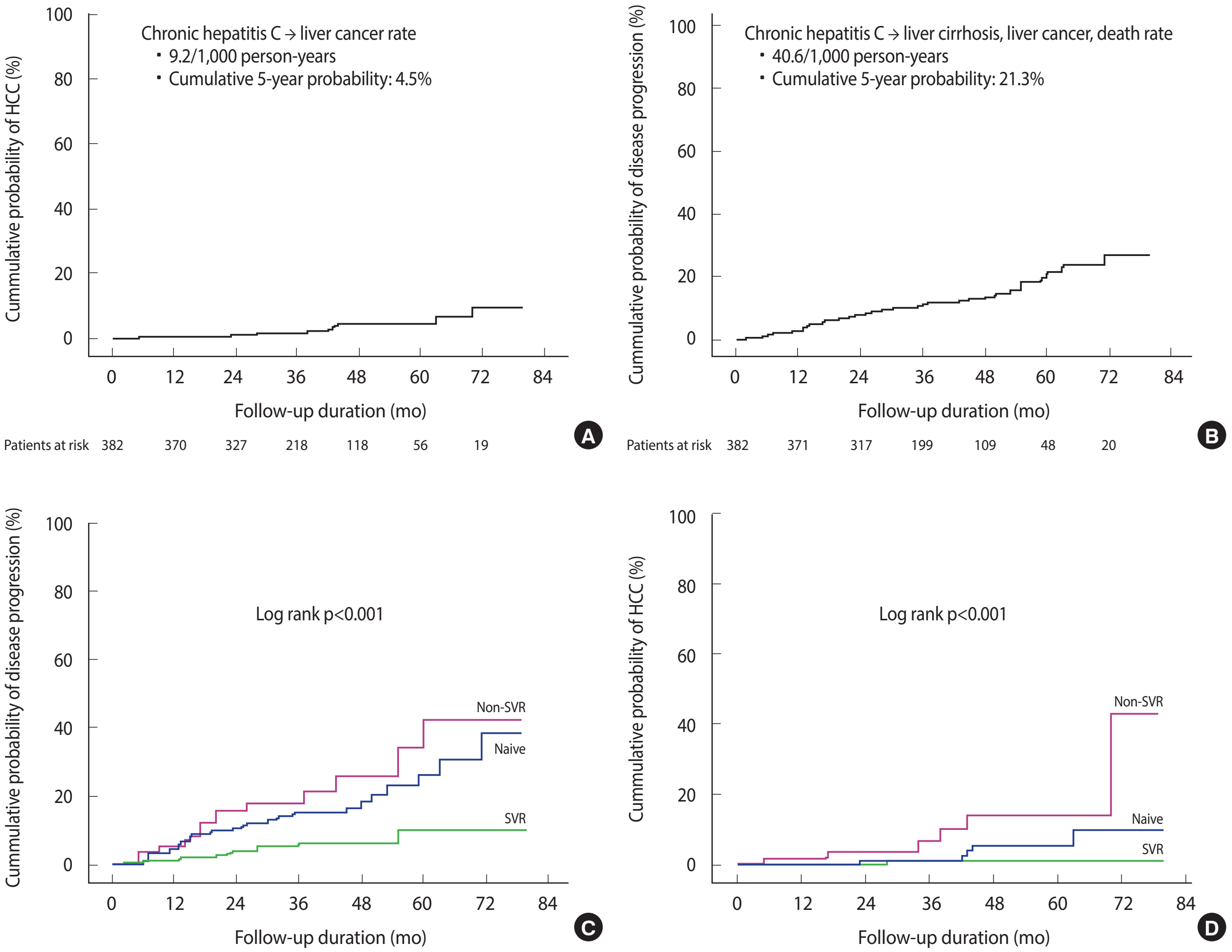

Figure 2. The prospective clinical outcomes of chronic hepatitis C patients in Korea (A and B) [5]. The difference of clinical outcomes of chronic hepatitis C patients among treatment-naive patients (Naive), those who did not achieve a sustained virologic response (Non-SVR), and those with a sustained virologic response (SVR). (C) The composite disease progression rate including development of liver cirrhosis, decompensation, liver cancer, and mortality was lowest in the "SVR" group compared to the "Non-SVR" or "Naive" group. (D) The cumulative probability of liver cancer development was lowest in the "SVR" group compared to the "Non-SVR" or the "Naive" group [5]. HCC, hepatocellular carcinoma.

터페론 기반의 항바이러스 치료는(페그인터페론 알파와 리바비린 병합치료로 유전자형 1형에서는 48주 치료하고 유전자형 2형에서는 24주 치료하는 요법) 전체 환자 중에서 37.3%에서 시도되었으며, 진단 후 5년 누적 항바이러스 치료율도 39.4%로 이 치료의 부작용이나 금기 등으로 약 2/3의 환자들이 치료받지 못함을 보여주었다[7]. 이 치료율은 환자와 의사가 항바이러스 치료에 가장 적극적인 경우, 즉 간전문가가 있는 대학병원의 결과인데 반해, 2009-2013년 건강보험심사평가원 자료를 분석한 결과에 의하면 5년간 총 340,756명 환자 중 35,006명만(10.3%) 인터페론 기반의 항바이러스 치료를 받아 실제 전국적인 항바이러스 치료율은 대학병원에서의 치료율의 1/3도 못 미칠 것으로 추정되었다[8].

경구용 C형간염치료제의 도입과 치료효과

페그인터페론 피하주사와 리바비린 경구투여의 병합요법은 HCV에 대한 비특이적 항바이러스 치료제였음에 반하여 2014년부터 사용되기 시작한 경구용 치료제는 HCV 생활사에 기반하여 HCV 증식의 주요 단계를 직접 차단하는 HCV에 특이적인 약제들이다(direct acting antivirals, DAA). 2015년부터 국내에서 승인되고 2016년부터 대부분 건강보험 급여적용을 받는 DAA는 Table 2에 요약하였다.

DAA 병합치료제는 경구약제를 12-24주 복용시 완치율이 90%를 상회하며 안전성도 탁월한 약제들로 과거 인터페론 기반치료의 대상이 되지 못하던 거의 모든 환자들이 DAA치료의 대상이

Table 2. Direct acting antiviral drugs against hepatitis C virus infection currently approved in South Korea

Drug Class	Drug	Manufacturer	Approved in Korea	
NS3/4A protease inhibitor	Asunaprevir	BMS	2015	Combination with daclatasvir
NS5A inhibitor	Daclatasvir	BMS	2015	Combination with asunaprevir or sofosbuvir
	Ledipasvir	Gilead	2015	Combination with sofosbuvir
NS5B inhibitor	Sofosbuvir	Gilead	2015	
NS5A/5B inhibitor	Ledipasvir/sofosbuvir	Gilead	2015	Single tablet complex

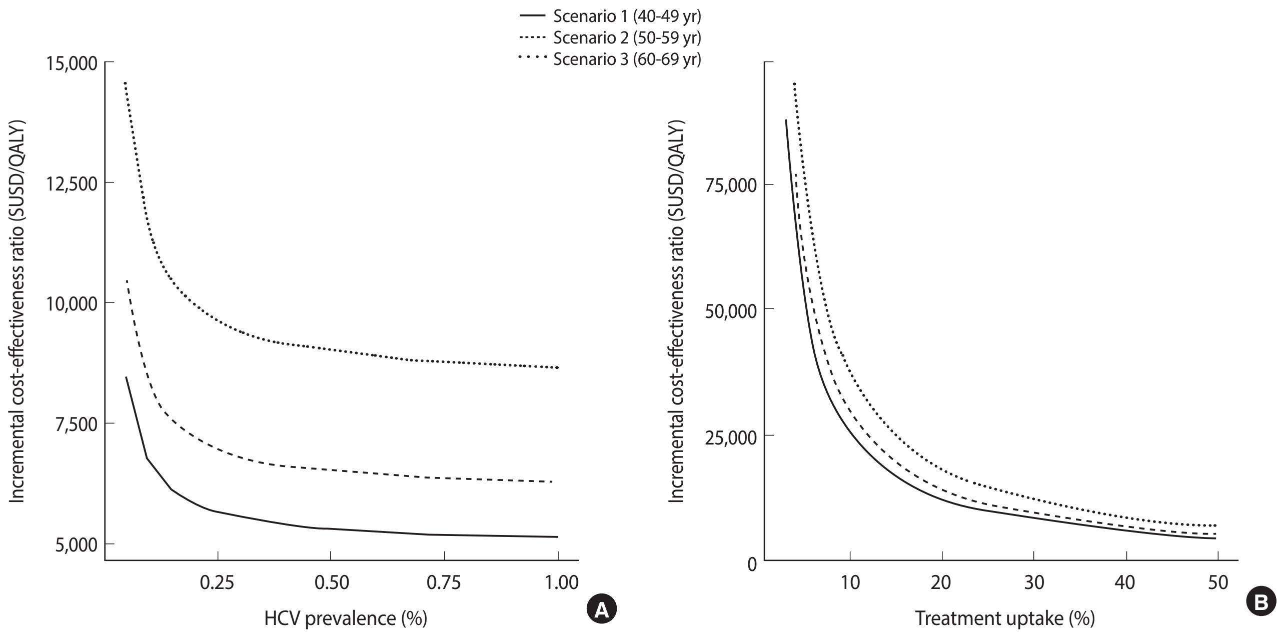

Figure 3. The cost-effectiveness analysis of anti-HCV screening for Korean population in their 5th, 6th, and 7th decade, respectively, with various prevalence (A) and treatment rates (B) [10]. QALY, quality adjusted life years.

된다[8]. DAA치료에 의한 HCV 퇴치라는 이상이 현실로 다가왔지만, 약제비용이 너무 높아 보험재정 및 환자들에게 경제적 부담을 주며, 약물상호작용에 유의하여야 하는 점 등이 치료에 걸림돌이 되고 있다. 또 국내 유병률 연구결과에서 언급한 바와 같이 아직 진단되지 않은 환자들이 많이 존재할 것이라는 점이 현재 국내 C형간염 관리의 중요한 화두가 되고 있다.

C형간염 국가적 관리체계 현황

2016년말 개정되기 전까지의 감염병 예방 및 관리에 관한 법률에 의하면 C형간염은 유행여부를 조사하고 감시해야 하는 지정감염병으로 분류되어 있었다. 2001-2010년까지 전국 보건의료원 및 병원급 이상 의료기관 대상에서, 2011년부터는 인구 20만명당 1개소의 병원급 이상 의료기관을(2016년, 186개소) 표본감시기관으로 지정하여 관할보건소에 신고하는 표본감시체계로 관리하였다[10]. 그러나 최근 집단감염 사태가 개인의원에서 주로 발생하여 표본감시체계에서 발견되지 않았던 점을 고려하여 2016년 9월 보건복지부의 C형간염예방 및 관리대책에는 전수감시 감염병 체계로 전환하겠다는 발표가 있었다. 이에 C형간염은 지정감염병에서 제3군 감염병으로 변경되었고 개정된 감염병예방법은 2017년 6월 3일부터 시행될 예정이어서, 추후 전수조사를 통한 적극적인 감염관리가 필요하다.

C형간염 선별검사의 필요성과 비용-효과성

2011-2012년 8개 대학병원을 방문한 445명의 C형간염환자들의 직접의료비용을 알아본 연구결과에 따르면 간질환 정도가 만성 C형간염에서 간경변증, 비대상성 간경변증, 간암으로 진행할수록 월간 환자별 평균 직접의료비용은 미화 183달러, 252달러, 1,020달러, 1,375달러로 증가하였다. 직접의료비용의 72%는 건강보험에서 지불되었고 28%는 환자 본인이 부담하였다. 평균 외래 방문일은 각 진단별로 7.01일, 7.36일, 8.30일, 11.56일이었고 입원일은 각각 0.19일, 0.77일, 8.75일, 12.86일이었다[10]. 만성간염 환자 중 항바이러스 치료로 완치된 환자군(69명)과 완치되지 않았던 환자군(215명)을 비교해본 결과 완치군의 평균 월별 진료비가 76달러임에 반해 완치되지 않은 군은 490달러로 크게 차이가 났다

[11]. 따라서 간경변증이나 간암으로 진행하기 전 단계에서 C형간염을 치료하는 것이 장기적인 관점에서 가장 비용효과적일 것이다.

한편 항바이러스 치료는 만성 C형간염 환자로 진단된 후에 이루어지는 일인데, 진단조차 되지 않은 환자군이 많이 존재한다는 증거가 있으므로 국내 인구대상 C형간염 선별검사의 필요성이 대두되었다. 미국에서는 anti-HCV 유병률이 높은 인구대상 선별전략이 비용효과적이라는 연구결과를 토대로 출생연도에 따른 선별검사가 시행되고 있다. 국내에서는 나이가 들수록 C형간염 유병률이 증가하므로 40대, 50대, 60대 인구를 대상으로 각각 1회 선별검사를 시행하고 현재 건강보험 급여기준에 합당한 DAA치료를 할 경우, 선별검사를 하지 않는 경우와 비교한 비용-효과연구 결과는 다음과 같았다. 이 전략을 사용할 경우 총 43,635명의 환자가 선별되고 17,193명이 치료받게 될 것으로 추정되었고, 40대, 50대, 60대 인구를 선별할 경우 각각 비용-효과증가비(incremental cost-effectiveness ratios, ICERs)는 질보정수명(quality-adjusted life year, QALY) 1년당 미화 5,714달러(840만 원), 6,843달러(1,076만 원), 8,889달러(1,589만 원)이었다(Figure 3). 국내에서 일반적으로 적용되는 지불의사금액 한계치(willingness-to-pay threshold, GDP per capita 2015)를 27,512달러로 가정할 경우, 40대, 50대, 60대 인구를 각각 1회 anti-HCV 선별검사를 시행하는 것은 비용-효과적이었으며, 특히 40대 인구 선별검사 전략이 가장 비용-효과적이었다[12].

우리나라는 전국민을 아우르는 검진체계가 갖춰져 있고, 검진기관수는 2012년말 8,576개소에 이른다. 2012년 일반검진 수검률은 72.9%, 암검진 수검률은 39.4%에 달하며, 건강보험공단이 지불한 검진비용은 약 9,672억이었다. 현재 국내 C형간염의 발생률은 알려지지 않았으나 수혈선별검사에 HCV RNA 검사가 시행되고 있고, 국내 정맥주사남용으로 인한 C형간염 발생률은 아직 낮은 편이다. 의료기관에서 발생하는 산발적 집단감염 사례에 대해서 최근 수립된 국가적 대응체계와 의사협회 차원의 의료기관 질관리 체계가 잘 운영된다면 향후 국내 C형간염의 발생률은 매우 낮을 것으로 추정된다. 따라서 향후 C형간염선별전략의 경제성 평가가 뒷받침된다면, 40-65세 인구에서 일반검진 검사 항목에 anti-HCV 검사를 생애 1회 추가하여 한시적으로 시행하고, 이후부터는 생애전환기 건강진단에 C형간염 항체검사를 포함하는 것을 고려해 볼 만하다. 2016년 9월 6일 보건복지부의 C형간염 예방 및 관리 대책 발표에서는 유병률이 높은 지역의 생애 전환기 건강진단 대상자들에서 C형간염 선별검사를 시범실시를 하겠다고 보도되었다.

맺는 말

현재 국내 인구의 C형간염 유병률은 1.0% 미만(0.6-0.8%)이며, 국내 수혈혈액 선별검사에 HCV RNA 검사를 시행하고 있어, 수혈에 의한 C형간염은 더 이상 발생하지 않을 것으로 생각된다. 그러나 최근 국내 의료기관에서 산발적 C형간염 집단감염이 발생하여 국가적 대응이 필요한 공중보건의 문제로 떠올랐다. 의료기관 및 비의료기관의 침습적 시술에 대한 엄격한 질관리를 국가적 차원에서 시행하고, 국민홍보를 통해 1회용 주사기사용 등에 대한 인지도를 높여야 한다. 한편 국내 정맥주사 약물남용으로 인한 C형간염 발생률은 아직 낮은 편이지만, 이를 차단하기 위해 정부 차원의 대응이 지속되어야 한다. 위에 열거한 항목들에 대한 종합적 예방대책이 수행되면 국내 C형간염의 발생률은 매우 낮아질 수 있을 것이다.

2016년부터 C형간염을 완치할 수 있는 효과적이고 안전한 약제가 건강보험급여로 인정되어 이미 진단된 환자들에서는 효과적인 치료가 시작되었다. 그러나 증상이 없거나 경미한 C형간염의 임상 특징으로 말미암아 진단되지 않은 상태에서 간질환이 진행되고 있는 환자들을 발굴하기 위해 국가 검진체계와 연계하여 선별검사를 시행한다면, 단기적으로는 진단과 치료비용 부담이 커지겠지만 장기적으로 사망률과 삶의 질을 높이는 비용효과적인 C형간염 퇴치 전략이 될 것이다. 시의적절한 대응과 관리전략을 통해 우리나라가 세계적으로 가장 빨리 C형간염 퇴치의 꿈을 이룰 수 있기를 기대한다.

감사의 글

이 연구는 질병관리본부 만성감염병 코호트 연구(Korea HCV Cohort study, 4800-4859-304)의 연구비와 2016년도 국립암센터 기관고유연구사업 (NCC-1410860) 지원을 받았음.

참고문헌

1. Kim DY, Kim IH, Jeong SH, Cho YK, Lee JH, Jin YJ, et al. A nationwide seroepidemiology of hepatitis C virus infection in South Korea. Liver Int 2013;33:586-594.
2. Shon HS, Choi HY, Kim JR, Ryu SY, Lee YJ, Lee MJ, et al. Comparison and analysis of the prevalence of hepatitis C virus infection by region in the Republic of Korea during 2005-2012. Clin Mol Hepatol 2015;21:249-256.
3. Seong MH, Kil H, Kim YS, Bae SH, Lee YJ, Lee HC, et al. Clinical and epidemiological features of hepatitis C virus infection in South Korea: a prospective, multicenter cohort study. J Med Virol 2013;85:1724-1733.
4. Sohn HS, Kim JR, Ryu SY, Lee YJ, Lee MJ, Min HJ, et al. Risk factors for hepatitis C virus (HCV) infection in areas with a high prevalence of HCV in the Republic of Korea in 2013. Gut Liver 2016;10:126-132.
5. Ok KS, Jeong SH, Jang ES, Kim YS, Lee YJ, Kim IH, et al. The clinical outcomes of chronic hepatitis C in South Korea: a prospective, multicenter cohort study. Medicine (Baltimore) 2016;95:e4594.
6. Lee SS, Jeong SH, Jang ES, Kim YS, Lee YJ, Jung EU, et al. Prospective

cohort study on the outcomes of hepatitis C virus-related cirrhosis in South Korea. J Gastroenterol Hepatol 2015;30:1281-1287.
7. Park SH, Park CK, Lee JW, Kim YS, Jeong SH, Kim YS, et al. Efficacy and tolerability of peginterferon alpha plus ribavirin in the routine daily treatment of chronic hepatitis C patients in Korea: a multi-center, retrospective observational study. Gut Liver 2012;6:98-106.
8. Jang ES, Kim YS, Kim KA, Lee YJ, Chung WJ, Kim IH, et al. Final report of unmet needs of interferon-based therapy for chronic hepatitis C in Korea: basis for moving into the direct-acting antiviral era. Gut Liver 2017. doi: https://doi.org/10.5009/gnl16530.
9. Korean Association for the Study of the Liver. KASL clinical practice guidelines: management of hepatitis C. Clin Mol Hepatol 2016;22:76-139.
10. Korea Centers for Diseases Control and Prevention. Year book 2015: national infectious diseases surveillance, Korea. Osong: Korea Centers for Diseases Control and Prevention; 2016, p. 531-532 (Korean).
11. Kim DY, Yoon KT, Kim W, Lee JI, Hong SH, Lee D, et al. Estimation of direct medical cost related to the management of chronic hepatitis C and its complications in South Korea. Medicine (Baltimore) 2016;95:e3896.
12. Kim DY, Han KH, Jun B, Kim TH, Park S, Ward T, et al. Estimating the cost-effectiveness of one-time screening and treatment for hepatitis C in Korea. PLoS One 2017;12:e0167770.
